# ApoE isoform leading to hypertriglyceridemia in new onset type 1 diabetes

**DOI:** 10.1002/ccr3.1584

**Published:** 2018-05-24

**Authors:** Christel Keefe, Sarah Lawson

**Affiliations:** ^1^ Cincinnati Children’s Hospital Medical Center University of Cincinnati Cincinnati OH USA

**Keywords:** adolescence, apolipoproteins, diabetic ketoacidosis, hypertriglyceridemia, hyponatremia

## Abstract

We report a case of pediatric diabetic ketoacidosis (DKA) with significant hypertriglyceridemia. The patient was found to have the e3/ e4 isoform of ApoE, increasing risk of hypertriglyceridemia in DKA. We suggest further genetic investigation for patients presenting with severe hypertriglyceridemia and DKA.

## 1 INTRODUCTION

Mild hypertriglyceridemia is often observed in patients who experience insulin deficiency in DKA.^1^ This results when the breakdown of triglyceride (TG) rich very low density lipoproteins (VLDL) and chylomicrons is interrupted. The key step in degradation of both chylomicrons and VLDL is lipoprotein lipase (LPL).^2^ The activity of LPL requires the presence of insulin and therefore, in insulin deficient and resistant states, the clearance of TG‐rich lipoproteins is impaired.^3^, ^4^ Another key aspect to lipid metabolism is the interactions between LPL and apolipoproteins. Apolipoproteins are located within the plasma membrane and facilitate lipid breakdown, transport, and metabolism. If altered, apolipoproteins can interfere with LPL activity which results in hyperlipidemia.

As stated above, mild hypertriglyceridemia (<11.3 mmol/L) is common in insulin deficiency; however, severe hypertriglyceridemia (serum triglycerides >16.9 mmol/L) is rare, especially in the pediatric population.^5^ When severe hypertriglyceridemia is seen in the setting of insulin deficiency, it should raise suspicion for an underlying disorder of dyslipidemia. Early detection of these disorders in childhood is important since correlations between lipid abnormalities, environmental factors, and early cardiovascular disease have been reported.^6^, ^7^ We review a case of an 11‐year‐old patient who presented with DKA in the presence of hypertriglyceridemia and concomitant electrolyte abnormalities.

## 2 CASE REPORT

An 11‐year‐old patient presented to his primary medical doctor (PMD) for a well‐child checkup. The patient was previously healthy, athletic, with no significant family medical history (pertinent negatives including a family history of diabetes, heart disease at a young age, hyperlipidemia, and hypertension). Outpatient documentation reported polyuria and polydipsia over 2 months, a 25 lb weight loss over 6 months, and new onset hyperglycemia with glycosuria. This prompted a referral to the emergency department (ED). On presentation to the ED, the patient was found to be tachycardic with normal systolic and diastolic blood pressures. His height was 150 cm (50th percentile), weight was 32.4 kg (10th percentile), and calculated BMI was less than the 5th percentile. He was prepubertal with unremarkable lung, heart, and abdominal examinations. Initial laboratory evaluation was significant for serum glucose of 26.8 mmol/L, sodium of 131 mmol/L (corrected for glucose to 135), bicarbonate of 12 mmol/L, and blood pH of 7.21. The patient was started on intravenous hydration (IVF) at 3000 mL/m^2^ ‐½ normal saline (NS) over 24 hours and an insulin infusion (0.1 U/kg/h) prior to transfer to the diabetes inpatient unit.

Euglycemia was achieved over the course of 6 hours but the patient remained acidotic (bicarbonate of 13 mmol/L). Therefore, IV insulin and dextrose containing fluids were continued. At the 10th hour of admission, serum sodium level was noted to fall to a nadir of 119 mmol/L (confirmed on repeat), despite the normalization of glucose concentration. Around the time of the sodium decline, the bedside nurse noted thick and milky serum. This prompted immediate evaluation showing cholesterol of 8.7 mmol/L (normal 4.0‐5.6), LDL of 2.2 mmol/L (normal 1.2‐3.1), HDL of 0.9 mmol/L (normal 0.7‐2.0), and TG of 19.8 mmol/L (normal 0.3‐1.5). The patient denied persistent abdominal pain and his lipase and amylase were normal. With this decrease in sodium, the patient remained stable without mental status changes. Hypertriglyceridemia and hyponatremia were managed with IVFs, which were transitioned to NS, and a decreased rate of insulin infusion (0.05 U/kg/h) to maintain glucose levels between 5.5 and 9.9 mmol/L. Acidosis improved (bicarbonate >18 mmol/L) within 16 hours. Sodium normalized by 18 hours. Prior to discharge, blood glucoses were stable and the TG level had trended downward to 13.0 mmol/L. A repeat lipid panel 2 weeks after discharge, in conjunction with well‐controlled glucose levels, showed cholesterol of 5.1 mmol/L and a TG of 0.4 mmol/L. There was no family history of type 1 diabetes, dyslipidemia, or coronary artery disease. Because of the abnormal hospital course, we sought to discover an explanation for the hypertriglyceridemia. Genetic isoform analysis preformed revealed to have the e3/ e4 isoform of ApoE.

## 3 DISCUSSION

We report a case of an adolescent with severe hypertriglyceridemia and pseudohyponatremia during an admission for DKA and new onset T1DM. Mild hypertriglyceridemia (<11.3 mmol/L) in DKA has been well described^4^, ^8^ and is a result of insulin deficiency, an essential component of proper LPL function. In studies, insulin replacement has been shown to significantly improve the activity of LPL in patients with insulin deficiency and leads to improved clearance of TG‐rich lipoproteins, namely chylomicrons and VLDL.^9^ Mutations in the LPL gene lead to severe (>16.9 mmol/L) and persistent hypertriglyceridemia in DKA that does not improve with insulin administration alone. This mutation is well‐documented, and in its homozygous form, often leads to chylomicronemia with severe (>16.9 mmol/L) hypertriglyceridemia, low HDL, and an increase risk of ischemic heart disease in both insulin deficient and sufficient states.^10^ Mutational analysis of the few reported cases of persistent and severe hypertriglyceridemia in DKA has mainly focused on LPL mutations.^1^, ^11^, ^12^ Severe, but not persistent, elevations in TG levels are rarely described in the literature and may be from abnormalities in lipoprotein cofactors, that is apolipoproteins. Apolipoproteins help package‐free cholesterol and TG to facilitate lipid transportation, storage, and metabolism. Patients with genetic alterations leading to the abnormal structure of apolipoproteins are known to have abnormalities in their lipid panels.^13^ Varying abnormalities in apolipoprotein classes lead to differing cardiovascular risk.^10^ To our knowledge, there are no previous reports of mutations in apolipoproteins in DKA in the pediatric population.

In our patient, testing for LPL mutations and other familial hyperlipidemia syndromes were not preformed given the resolution of severe hypertriglyceridemia after the correction of DKA, the lack of continued hypertriglyceridemia or hyperlipidemia with insulin therapy alone, and a negative family history for hyperlipidemia. Therefore, analysis was sent on the cofactor which is seen on all triglyceride‐rich lipoproteins, ApoE. ApoE is found on chylomicrons, VLDL, low (LDL) and high density lipoproteins (HDL) and has an affinity for the low density lipoprotein receptor which allows uptake of lipoproteins by the liver.^13^, ^14^ Furthermore, ApoE is a modulator of LPL‐mediated hydrolysis of TGs on muscle and fat tissue.^14^ There are 3 isoforms of Apo E present in the human population; Apo E3, E4, and E2, their allele frequencies are ~0.75, 0.15, and 0.10, respectively.^15^ The common ApoE isoforms perform the above functions to varying degrees of effectiveness resulting in differing risks of cardiovascular disease.^15^ ApoE3 is the most efficient in aiding the breakdown of TGs, leading to the lowest cardiovascular risk.^13^ ApoE4 binds strongest to LPL when on the surface of VLDL; ultimately, impairing breakdown of VLDL. This in turn leads to a higher VLDL to HDL ratio which is pro‐atherogenic.^13^ This ultimately leads to carriers of the ApoE4 allele having a twofold increase in their mortality risk, as found in one study of myocardial infarction survivors.^16^ ApoE2 exhibits impaired binding of lipoproteins to the LPL receptor which leads to a decreased clearance of TG‐rich lipoproteins. In a homozygous form, if combined with additional genetic or environmental factors, ApoE2 leads to overt hyperlipidemia, otherwise known as familial dysbetalipoproteinemia.^13^, ^15^ The patient was found to have the e3/ e4 isoform of ApoE. This isoform has been associated with many clinical findings related to obesity^17^ and neurodegenerative disorders^18^ in addition to an increased cardiovascular risk in the general population^10^ and increase carotid artery intima‐media thickness in T1DM.^14^ Of crucial importance in our patient was the association between the e3/e4 isoform of ApoE and decreased clearance of chylomicrons and VLDL.^19^ There is a direct correlation between Apo E3/4 and increased LDL C and coronary artery risk.^19^ When this was combined with decreased LPL activity, from insulin deficiency, severe hypertriglyceridemia resulted. Given resolution of hypertriglyceridemia once insulin was administered and glycemic control achieved, treatment recommendation for this patient was limited to a diet low fat and high in fiber as well as daily activity. The patient and family were also counseled that poor diabetes control would likely lead to elevated triglycerides and potential for pancreatitis. Further, given that carriers of the epsilon4 isoform have a slightly higher cardiovascular risk,^19^ counseling on healthy diet and activity was also recommended. Defects in Apo proteins can increase cardiovascular risk and therefore significant and or persistent elevations in triglycerides, in conjunction with DKA, should warrant further exploration into the cause. Understanding when an abnormality in lipid metabolism should be questioned will decrease unnecessary screening, thereby improving the cost/benefit ratio of testing, as most individuals with DKA have some degree of hyperlipidemia.

Along with hypertriglyceridemia, the patient was also noted to have extreme hyponatremia that was not explained by the degree of hyperglycemia or hypertriglyceridemia. Hyponatremia is common in insulin deficiency and is due to hyperglycemia osmotically drawing water into the vascular space. This type of hyponatremia is considered artifactual and can be adjusted for with a simple equation. The patient had hyponatremia which worsened overnight to a nadir of 119 mmol/L despite correction of hyperglycemia. This degree of hyponatremia was not explained by hyperglycemia alone. A literature search revealed that hypertriglyceridemia associated with hyponatremia and DKA has been described in at least one other case report. In that previous report, the etiology of this significant degree of hyponatremia was unknown and the hyponatremia corrected with the administration of IV 3% NaCl solution.^20^ In our patient, close observation and a change in the sodium concentration in the IVF’s (1/2NS to NS) resulted in resolution of the perceived hyponatremia. The etiology of severe hyponatremia is still unknown, but thought to be a dilution effect. It is important that, the patient was not noted to have clinical manifestation of hyponatremia, that is mental status alteration or seizure, and the abnormality resolved with minor intervention. Hyponatremia occurs in DKA and could be worsened by hypertriglyceridemia. Patients with the conjunction of these 2 conditions should be monitored very closely, that is neurologic examinations and laboratory evaluation. To our knowledge, this is the first case report of hypertriglyceridemia in DKA associated with an ApoE polymorphism, this presentation was also complicated by hyponatremia.

## 4 CONCLUSION

This is a case demonstrating electrolyte abnormalities and significant hypertriglyceridemia in a patient with new onset T1DM in DKA. We speculate that the genetic isoform ApoE3/4 leads to elevated triglyceride levels during a state of insulin deficiency that had led to LPL insufficiency. Hypertriglyceridemia and hyponatremia resolved without significant intervention, mainly correction of DKA, and the patient remained stable without mental status changes. Mild hypertriglyceridemia is common in DKA, but in patients with lipemic serum and significant elevations in triglycerides (>16.9 mmol/L), one should consider further screening for lipid metabolism and structural abnormalities.

## CONFLICT OF INTEREST

None declared.

## AUTHORSHIP

CK: compiled the data, performed primary drafting of the manuscript, reviewed and revised the manuscript. SL: reviewed and revised the manuscript.
